# Wearable Sensor Fusion for Psychophysiological Arousal Recognition in Air Traffic Control: A Fuzzy MCDM Approach

**DOI:** 10.3390/s26196272

**Published:** 2026-10-03

**Authors:** Manal Aich, Jamal El Aoufi, Ahmed Moussa

**Affiliations:** 1 Systems and Data Engineering Team, National School of Applied Sciences-Tangier, Abdelmalek Essaadi University, Tétouan 93000, Morocco; amoussa@uae.ac.ma; 2 Department of Air Traffic Control, Mohammed VI International Academy of Civil Aviation, Nouaceur 27000, Morocco; j.elaoufi@onda.ma

**Keywords:** emotion recognition, affective computing, physiological signal analysis, multi-modal data fusion, wearable devices, sensor technologies, real-time monitoring, human–computer interaction, fuzzy multi-criteria decision making, air traffic control, vigilance, machine learning

## Abstract

Recognizing psychophysiological arousal and strain from wearable sensors extends affective computing beyond discrete-emotion classification. Vigilance decrement during air traffic control (ATC) shifts illustrates this: the psychomotor vigilance task (PVT) cannot be administered while a controller is working. We develop and evaluate a wearable, label-free Vigilance/Strain Risk Index (F-VRI) for ATC operators, fusing five physiological signals via entropy-weighted fuzzy TOPSIS. The index was assessed in two samples: 38 controllers measured pre/post shift (preliminary, pilot-level evidence) and 13 trainees pre/post across simulator sessions (confirmatory validation). Compared with PVT, the fused index detected the pre/post change more reliably at the group level (simulator: *p* = 0.004 vs. *p* = 0.379) and at the individual level (68–80% directional concordance vs. 33–55% for PVT, at or below chance). Compared with five supervised machine learning baselines, the fuzzy index was the only method to remain significant under cross-domain transfer (both *p* < 0.05), while every supervised model failed in at least one direction. An exploratory extension revealed a weighting pathology in which static covariates absorbed disproportionate model weight, which led us to a two-layer state/trait architecture. These results support fuzzy MCDM as an interpretable sensor-fusion framework for psychophysiological state monitoring under uncertainty, with continuous, streaming deployment remaining a prospective extension requiring further validation; the field-cohort effect (*p* = 0.084) remains at the pilot level.

## 1. Introduction

Maintaining operator vigilance is a foundational safety requirement in air traffic control (ATC), where a single missed conflict or delayed response can have catastrophic consequences. Vigilance is not a stable trait, however: sustained-attention research dating to Mackworth’s [1] classic clock-watching studies has repeatedly shown that the ability to detect infrequent, safety-critical signals deteriorates measurably over time on task, a phenomenon termed the vigilance decrement. In monotony-dominated monitoring tasks, of which low-traffic ATC sectors and overnight shifts are canonical examples, this decrement is generally attributed to an underload or resource-depletion process rather than to overt fatigue from workload overload [2]. This distinction matters for how a continuous monitoring system should be designed and interpreted, and the paper returns to it in Section 5.4.

Vigilance decrement, operator strain, and workload-related arousal are instances of a broader class of problems addressed by sensor-based emotion recognition and affective computing: the inference of a person’s internal psychophysiological state from continuously sampled physiological signals rather than from self-report or overt behavior [3]. Classical affect models such as the circumplex distinguish an arousal dimension (physiological activation, indexed by autonomic markers such as heart rate and heart rate variability) from a valence dimension (positive-to-negative hedonic tone); autonomic nervous system activity of the kind targeted here has long been understood as a core physiological substrate of this arousal dimension [4]. Over the past three years, wearable-sensor-based affective computing has shifted from laboratory emotion-elicitation paradigms to continuous, everyday- and workplace-relevant state recognition using consumer-grade wristband sensors [3,5,6]. Cardiac signals specifically have proven a rich substrate for this shift: a photonic sensing system decoding seismocardiography signals has recently been shown to extract subject-invariant emotional information via complex-network feature representations, directly targeting the inter-subject variability that has historically limited cross-subject generalization in cardiac-signal-based affective computing [7]. The present study can therefore be read as a safety-critical application of this wider arousal/strain-recognition problem, in a domain, ATC, where the operational cost of an undetected state change is unusually high and where interpretability and auditability of the recognition system are not optional design preferences but operational requirements.

The behavioral gold standard for quantifying vigilance is the psychomotor vigilance task (PVT), a sustained-attention reaction-time test whose sensitivity and specificity to fatigue-related performance decline have been extensively validated in controlled sleep-deprivation studies [8,9], and a 2025 field study applying the brief PVT-B directly across ATC shift types confirms this sensitivity extends to operational area-control settings [10]. Its central operational limitation is precisely what makes it psychometrically useful: it requires the operator to disengage from their working task and dedicate several uninterrupted minutes to the test itself. This is incompatible with continuous, real-time monitoring during live ATC duty, where a controller cannot be asked to stop scanning traffic to complete a reaction-time test. In practice, this restricts PVT to intermittent, intrusive administration, before and after a shift or during scheduled breaks, which leaves most of an operational shift unmonitored.

Wrist-worn wearable devices offer a non-intrusive alternative: heart rate, heart rate variability (HRV), peripheral oxygen saturation, respiratory rate, and device-derived stress scores can, in principle, be sampled continuously without interrupting task performance. Physiological correlates of workload and strain have been documented in ATC and related operational-monitoring settings using heart rate and HRV [11], feeding growing interest in wearable-based, continuous operator-state monitoring as an operationally feasible complement, or alternative, to intermittent behavioral testing. The same signals, and the same continuous-monitoring rationale, underlie the broader wearable-sensor affective-computing literature reviewed in Section 2.6, which has established heart rate, HRV, electrodermal activity, and respiration as the dominant modalities for physiological-signal-based emotion and stress recognition [3,5,6,12,13,14].

Four gaps still limit the practical translation of prior work into a deployable ATC vigilance-monitoring tool. First, most vigilance-modeling studies validate a proposed measure in a single context only (either a controlled laboratory or simulator setting, or a field/operational setting, but rarely both), which leaves open whether findings generalize from the conditions under which a model is built to the conditions under which it would actually be deployed; this single-context limitation is echoed in recent affective-computing reviews, which flag the scarcity of studies that validate a wearable-derived state-recognition model across meaningfully different deployment contexts [3,6]. Second, the analytic approaches typically applied to these signals sit at two extremes: classical inferential statistics on raw physiological measures, which do not naturally fuse multiple heterogeneous criteria into a single decision-relevant index, or supervised machine learning models, which can fuse multiple inputs but require substantial labeled training data and behave as comparatively opaque black boxes. Neither is a good match for the small-sample, label-scarce, interpretability-constrained conditions that characterize real ATC operational safety research, where labeled fatigue events are rare, expensive to obtain, and ethically constrained. Third, fuzzy multi-criteria decision-making (MCDM) methods are already well established as an auditable, label-free framework for multi-criteria risk ranking in road traffic safety [15,16], and have recently begun to be applied directly to physiological driver-stress recognition using fuzzy and interval type-2 fuzzy logic [17,18], offering a plausible solution to both problems, since they require no outcome labels to construct and produce a transparent, rule-legible risk score. Fourth, sensor-fusion architectures for continuous affective- or strain-state monitoring are increasingly expected to operate within an Internet-of-Things (IoT) deployment model, streaming from a wearable or in-vehicle sensor node to a real-time scoring pipeline [12,19]; a fuzzy MCDM index of the kind developed here is directly compatible with this deployment model, since it requires no retraining and produces an explicit, low-latency risk score from a fixed set of criteria. To the authors’ knowledge, however, this paradigm has not previously been evaluated across both a simulator and a live-operational ATC setting, nor benchmarked directly against supervised machine learning under an explicit cross-domain transfer test, nor framed explicitly within the wearable-sensor affective-computing literature to which it is methodologically closest.

This paper addresses these four gaps directly. We develop a Fuzzy Vigilance/Strain Risk Index (F-VRI) using entropy-weighted fuzzy TOPSIS to fuse five continuously wearable-derivable physiological criteria into a single, label-free vigilance/arousal risk score, and evaluate it in two independent cohorts spanning a live-operational field setting and a simulator-based training setting, with confirmatory validation achieved in the simulator cohort and preliminary, pilot-level feasibility evidence in the field cohort (Section 6). We benchmark the index directly against the PVT behavioral standard and against five supervised machine learning baselines, under both a best-case within-domain protocol and an explicit cross-domain transfer test that, to our knowledge, is rarely reported in this literature. We further document, and structurally correct, a weighting pathology that arises when static between-person covariates are pooled with dynamic physiological criteria under a single entropy-weighting scheme. The remainder of the paper is organized as follows: Section 2 reviews the relevant vigilance, physiological-monitoring, fuzzy MCDM, machine learning, and wearable-sensor affective-computing literature; Section 3 describes the two study cohorts and the fuzzy index construction; Section 4 reports the validation results; Section 5 discusses their interpretation and relation to prior work; Section 6 addresses limitations; and Section 7 concludes.

## 2. Related Work

This section situates the present study within six bodies of prior work: the psychological theory of vigilance decrement (Section 2.1); physiological correlates of fatigue and workload in ATC settings (Section 2.2); the psychomotor vigilance task as behavioral gold standard (Section 2.3); fuzzy multi-criteria decision making in transportation safety and physiological state recognition (Section 2.4); machine learning approaches to fatigue/vigilance classification (Section 2.5); and wearable-sensor affective computing and emotion recognition (Section 2.6).

### 2.1. Vigilance Decrement and Resource Theory

The empirical foundation of vigilance research is Mackworth’s [1] demonstration that operators monitoring a simulated radar-type display for infrequent signals showed a marked decline in detection accuracy within the first 30 min of a prolonged watch, a finding replicated across countless subsequent paradigms and now generally termed the vigilance decrement; a 75-year retrospective on this literature confirms the decrement remains one of the most robust and actively studied phenomena in attention research [20]. Two broad theoretical accounts have been offered for this decline. Arousal-based accounts attribute the decrement to a decline in general physiological arousal over time on task. Resource-based accounts, developed most extensively by Warm et al. [2] and refined in recent theoretical, contextual, and neural syntheses [21], instead characterize sustained attention as an inherently effortful, resource-depleting activity: vigilance, in this view, is not a passive, low-demand state but one that is subjectively stressful and cognitively costly to maintain, such that performance and self-reported strain both degrade as available attentional resources are consumed. A related underload or mindlessness account instead proposes that operators disengage from monotonous, low-event-rate tasks rather than actively depleting a resource pool. These accounts are not mutually exclusive, and the appropriate account may depend on task demand: high-workload, information-rich monitoring is more consistent with an overload/resource-depletion profile, whereas low-event-rate, monotonous monitoring, closer to the profile of a quiet ATC sector or an overnight shift, is more consistent with an underload account. This distinction is directly relevant to the field-cohort finding reported in Section 4.1 of declining physiological arousal indicators without a corresponding group-level PVT decline, a pattern discussed further in Section 5.4.

### 2.2. Physiological Correlates of Fatigue and Vigilance in ATC Settings

Physiological monitoring of ATC operators has a long history in the human factors and ergonomics literature, motivated by evidence that operator state contributes materially to safety-relevant performance variation. Heart rate and heart rate variability measures in particular have been used to index workload and autonomic strain in simulated and operational ATC tasks: both track the balance of sympathetic and parasympathetic nervous system activity under varying traffic load and task demand [11], a pattern corroborated by more recent case-study evidence linking ATC rostering, sleep quality, vigilance, and workload directly [22]. This body of work establishes HR and HRV as viable, non-intrusive workload and strain indicators in ATC-relevant settings, but has generally focused on group-level workload contrasts (for example, comparing shifts or traffic-density conditions) rather than on constructing an individual-level, continuously updatable vigilance risk score suitable for real-time operator monitoring, which is the specific gap addressed by the present study’s fusion of HR, HRV, SpO_2_, respiratory rate, and a device-derived stress score into a single index.

### 2.3. The Psychomotor Vigilance Task as Behavioral Gold Standard

The psychomotor vigilance task, in its standard 10 min form and validated shorter 5 and 3 min variants, is widely regarded as the de facto behavioral gold standard for quantifying fatigue-related decrements in sustained attention, owing to extensive psychometric work establishing which PVT metrics and durations most reliably discriminate sleep-deprived from rested individuals [8,9]. Its validity is well established; its practical limitation, discussed in Section 1, is operational rather than psychometric: a task requiring several minutes of dedicated, uninterrupted attention cannot be embedded within live ATC duty without displacing the very vigilance it is meant to measure. This tension between measurement validity and operational deployability motivates the present study’s treatment of PVT strictly as an offline validation benchmark (Section 3.5) rather than as a candidate for the deployable index itself.

### 2.4. Fuzzy Multi-Criteria Decision Making in Transportation Safety and Physiological State Recognition

Fuzzy set theory [23], combined with multi-criteria decision-making methods such as the Analytic Hierarchy Process (fuzzy AHP) and the Technique for Order of Preference by Similarity to Ideal Solution (fuzzy TOPSIS) [24], has been applied extensively to road traffic safety problems, including ranking the factors most strongly associated with accident rates [15] and prioritizing hazard locations for road-safety intervention using integrated fuzzy AHP-TOPSIS frameworks [16]. These applications share three properties that motivate their extension to ATC operator monitoring: they require no outcome labels to construct, since criterion weights are derived either from expert elicitation or, as in the entropy-weighting approach adopted here, directly from the objective variance structure of the data; they produce an auditable, rule-legible risk score rather than an opaque model output, a property of particular importance in safety-critical decision support; and they handle the imprecision inherent in continuous physiological or behavioral measurement through fuzzy membership functions rather than requiring hard, potentially arbitrary thresholds.

Within air traffic control specifically, non-wearable multimodal sensor fusion has already been used for controller fatigue detection: a fuzzy-fusion ensemble combining facial and vocal features [25], and, more recently, a deep dual-stream convolutional network fusing radiotelephony audio with facial video [26], both report strong within-sample fatigue-detection accuracy without requiring the controller to interrupt task performance. These studies confirm that fusion-based, PVT-free ATC operator-state detection is an active research direction, and motivate the present study’s parallel case for a wearable-physiological, rather than facial/vocal, fusion modality.

Road traffic safety is no longer the only domain in which this fuzzy MCDM track record has been built. A closely related and much more recent literature has applied fuzzy and interval type-2 fuzzy logic directly to physiological driver-stress recognition: a fuzzy-based driver stress detection system using an interval type-2 fuzzy logic system to fuse HRV, galvanic skin response, steering-angle variation, and traffic density into an interpretable, uncertainty-aware stress level [17]; a fuzzy performance-estimation framework for evaluating real-world driver-stress recognition models built on physiological signals and deep learning [18]; and an Internet-of-Things-oriented in-vehicle monitoring system that fuses wearable heart rate and thermal-imaging data to assess driver arousal in real time [19]. These studies are, in essence, the same problem addressed here, physiological, sensor-fused, uncertainty-aware recognition of an operator’s arousal/strain state, applied to a structurally analogous safety-critical, human-in-the-loop traffic-management domain. Given this structural similarity between road/vehicle traffic management and ATC, it is notable that the authors are not aware of a prior fuzzy MCDM application that has been cross-validated between a simulator and a live-operational ATC vigilance-monitoring setting, the specific extension undertaken here. More broadly, fuzzy MCDM has continued to expand well beyond transportation into other safety- and health-relevant continuous-monitoring domains: entropy- and Fermatean-fuzzy variants of TOPSIS/EDAS have recently been used to select wearable sensors for remote patient monitoring [27], and fuzzy decision-support systems have separately been applied to real-time physiological fatigue monitoring in athletes [28], both sharing the same label-free, auditable rationale that underlies the present ATC application; a recent two-decade bibliometric review of the MCDM literature documents this broader shift toward hybrid, uncertainty-aware decision frameworks across domains [29].

### 2.5. Machine Learning for Fatigue/Vigilance Classification and the Generalization Problem

A separate literature has applied supervised machine learning to physiological-signal-based fatigue and drowsiness classification, predominantly in driving contexts, and a 2024 review confirms that AI-based fatigue and sleep-problem detection is now a rapidly expanding research direction across aviation specifically, spanning pilots and controllers alike [30]. Wearable HRV-derived features have been used with classifiers including support vector machines, random forests, and other standard supervised algorithms to detect drowsiness from photoplethysmography and electrocardiography signals [31], typically reporting strong within-sample or within-domain classification performance. However, this literature consistently faces two constraints directly relevant to the present study: sample sizes are often small relative to model complexity, and cross-domain or cross-subject generalization (training a model in one setting or population and testing it, without retraining, in another) is rarely evaluated, despite being the more realistic test of deployment readiness for any monitoring system intended to generalize across operational contexts. Where cross-domain or cross-subject transfer has been directly tested, performance degradation relative to within-domain evaluation is a common finding, consistent with model overfitting to domain-specific noise under label-scarce conditions. This motivates the present study’s inclusion of an explicit cross-domain transfer test (Section 3.6) as a necessary, rather than optional, complement to within-domain model comparison, and its comparison of the proposed label-free fuzzy index directly against supervised baselines under both protocols (Section 4.3).

### 2.6. Wearable-Sensor Affective Computing and Emotion Recognition

The present study is methodologically closest to the fast-growing literature on emotion and affect recognition from wearable physiological sensors, which shares its central premise, that continuously sampled autonomic signals carry decodable information about a person’s internal state, without requiring behavioral interruption or explicit self-report at inference time. A 2023 systematic literature review of emotion recognition for everyday life using wearable physiological signals catalogued the dominant modalities (heart rate, HRV, electrodermal activity), the recurring pipeline of stimulus elicitation, self-report labeling, and supervised classification, and identified the transition from controlled laboratory elicitation to in-the-wild recognition as the field’s central open challenge [3]. More recent work has pushed further into everyday, multi-day, naturalistic recording: a 2025 study used a transformer architecture to recognize positive/negative and valence-arousal affective states from five-day continuous wrist-worn recordings of heart rate, skin conductance, and acceleration, explicitly citing its relevance to human–computer interaction and psychiatric monitoring applications [5]; a companion 2025 systematic review of multimodal physiological wearable sensing for affective computing found that electrodermal activity and electrocardiography remain the dominant modalities and that deep architectures (CNNs, LSTMs, and increasingly transformers) dominate the modeling literature, while flagging the continued scarcity of long-term, continuous, naturalistic-setting data as a central limitation, a limitation the two-cohort, pre/post design of the present study addresses only partially but directly acknowledges (Section 6) [6]. A parallel 2025 systematic review of open physiological-signal stress datasets similarly reports that multimodal fusion, personalized and adaptive modeling, and explainable methods dominate the 2023–2025 methodological literature on stress-state recognition specifically [13], and a 2025 review of Internet-of-Things- and AI-enabled real-time stress detection and modulation situates this same recognition problem within a deployment architecture, streaming wearable and mobile-sensor data through AI models for continuous, real-world monitoring, that is structurally identical to the deployment pathway envisioned for the present index in Section 5.5 [12].

Two features distinguish the present contribution from this broader affective-computing literature rather than simply situating it within one. First, essentially all of the work just reviewed relies on supervised or deep-learning classifiers trained against self-reported or externally annotated emotion or stress labels; the present study instead targets a domain, live operational ATC, in which continuous ground-truth labeling is operationally infeasible and ethically constrained; this is why we develop the label-free fuzzy MCDM approach here as a direct alternative to the supervised paradigm that dominates wearable affective computing. Second, almost none of the reviewed studies report a genuine cross-context or cross-population transfer test of the kind performed in Section 3.6 and Section 4.3; the present results (Section 4.3) suggest that this omission may be masking a real generalization gap in supervised wearable affect-recognition pipelines more broadly, not only in the ATC vigilance application studied here. The present work can therefore be read both as a safety-critical case study in physiological arousal/strain recognition and as evidence, relevant to the broader sensor-based emotion-recognition field, that label-free, auditable fusion methods deserve more systematic comparison against the now-dominant supervised deep-learning paradigm, particularly under cross-domain transfer.

## 3. Materials and Methods

**Figure 1:**
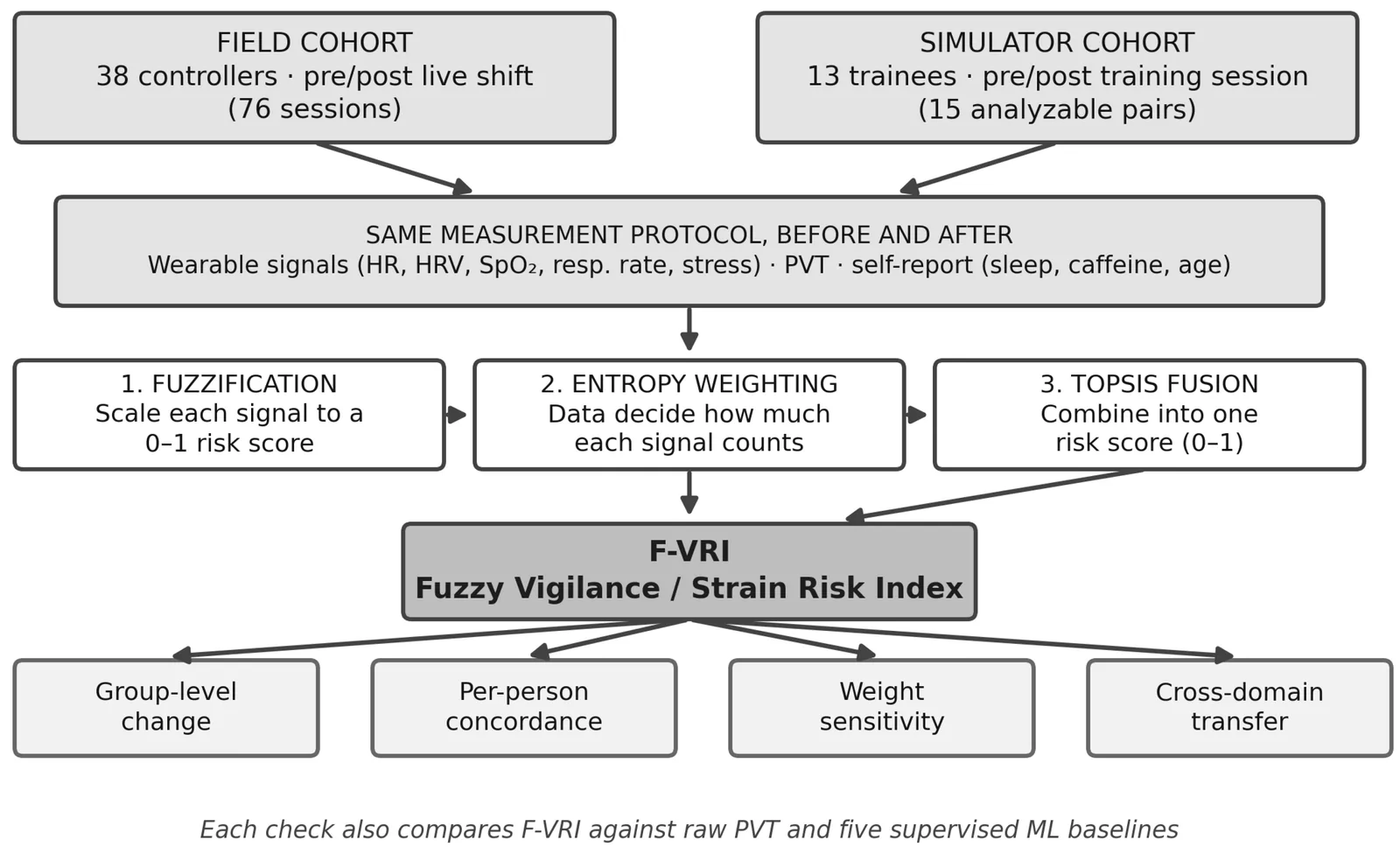
Overall study workflow: two cohorts, one measurement protocol, one fuzzy-index pipeline, four validation checks. Shading is used only to group elements: medium gray, data collection (the two cohorts and the shared measurement protocol); white, the three index-construction steps; dark gray, the resulting F-VRI index; light gray, the four validation checks.

Figure 1 summarizes the overall study workflow, from the two cohorts and the shared measurement protocol, through the three-step fuzzy index pipeline, to the four validation checks described in Section 3.5.

### 3.1. Participants and Study Design

Two cohorts contributed data. The calibration cohort comprised 38 professional air traffic controllers, each measured once before and once after an operational session (76 total sessions). The validation cohort comprised 13 ATC trainees tested in a simulator environment, contributing 30 sessions in total (most trainees contributed two sessions; two trainees contributed four sessions each, corresponding to repeated pre/post measurement blocks).

The 13 simulator-cohort trainees volunteered to participate while in the simulator phase of their air traffic control training program. Over a 15-day study period, each trainee completed a 3 min PVT immediately before, and again immediately after, each simulation session. Before each session, participants self-reported their sleep duration over the preceding night and their caffeine intake since waking, and heart rate, heart rate variability, respiratory rate, peripheral oxygen saturation, and a device-derived stress score were recorded via a wrist-worn Garmin Forerunner 255 (Garmin Ltd., Olathe, KS, USA). The same protocol, self-report questionnaire, 3 min PVT, and wearable physiological recording, was repeated immediately after each session. Age was recorded once, at study entry, since it does not vary within a trainee across sessions. The 38 aerodrome air traffic controllers of the field cohort volunteered to participate during their live operational shifts and followed the same measurement protocol, administered immediately before and immediately after their shift. Full details of the ethics review and participant consent are reported in the ethics and informed consent statements at the end of the article.

The two cohorts differ along several dimensions beyond measurement setting (simulator vs. live operations), which the terms “cross-domain” or “domain transfer” used elsewhere in this paper (Section 3.6 and Section 4.3) should be understood as shorthand for, rather than as isolating environment alone. Professional controllers and trainees plausibly differ in expertise, age, task demands, stress exposure, task predictability, motivation, baseline autonomic state, and possibly session duration; the present dataset cannot separate these confounded dimensions from one another. The cross-domain transfer test (Section 3.6 and Section 4.3) is for this reason best read as a joint cross-population/cross-context stress test of the fitted models, not as isolating simulator-to-field environmental transfer specifically; we adopt the more precise term cross-population/cross-context transfer when interpreting these results (Section 5.2), and retain “cross-domain transfer” only as an established, concise label for the train-on-one-test-on-the-other methodology itself (Section 3.6).

The two trainees contributing four sessions (two independent pre/post blocks) are treated as contributing two independent pre/post pairs each for the paired analyses reported below, yielding 15 analyzable pre/post pairs from 13 trainees. This introduces a minor non-independence for the two repeated trainees, addressed as a limitation in Section 6.

A mixed-effects model was considered but not used as the primary analysis, since only 2 of the 13 trainees contribute a repeated pre/post block, too few clusters to estimate a subject-level random effect reliably. As a sensitivity check, excluding these two trainees entirely (11 independent pairs) preserved the significant pre/post effect (*p* = 0.024, dz = −0.77, 72.7% concordance), as did retaining only their first pre/post block (13 independent pairs; *p* = 0.006, dz = −0.94, 84.6% concordance), indicating the simulator-cohort result reported in Section 4.2 is not an artifact of the repeated-measures pairs.

### 3.2. Measures

Vigilance (ground truth) was measured with the PVT, expressed as mean reaction time in milliseconds; higher values indicate poorer vigilance. Physiological criteria, heart rate, heart rate variability, peripheral oxygen saturation, respiratory rate, and a device-derived physiological stress score, were recorded with a Garmin Forerunner 255 wrist-worn device; these are the same class of autonomic, arousal-linked signals used throughout the wearable-sensor affective-computing literature reviewed in Section 2.6. Behavioral and demographic criteria, self-reported sleep duration (converted to decimal hours), caffeine dose (converted to milligrams using a standard reference table for common beverages), and age, were collected by self-report prior to each session; sleep, caffeine, and reaction-time relationships in this same Moroccan ATC population have been examined separately in a companion analysis [32].

In the simulator cohort, the Karolinska Sleepiness Scale (KSS) [33], a 9-point subjective sleepiness rating, was additionally collected and used exclusively as an offline validation criterion (Section 3.5); it was never used as a model input.

KSS was not administered in the field cohort because controllers on live operational duty could not be asked to complete an additional self-report instrument around a shift without disrupting operational constraints, whereas the simulator training protocol had scheduled time available for it. This means KSS-based construct validity (Section 4.2) can be assessed only in the simulator cohort; PVT, collected identically in both settings, remains the sole construct-validity anchor common to both cohorts.

Each physiological reading (HR, HRV, SpO_2_, respiratory rate, stress score) was captured via the device’s built-in ~2-min spot-check acquisition (Garmin’s Health Snapshot-style feature), rather than a longer continuous averaging window; each value therefore reflects a brief, device-aggregated snapshot at the pre- or post-session timepoint rather than an instantaneous single-sample reading. Missing or clearly erroneous readings (e.g., a failed spot-check acquisition) were handled case-by-case rather than through a fixed automated rule; no additional signal-quality threshold beyond the device’s internal acquisition-quality gating was applied by the study team. Consumer-grade wearable-derived HR, HRV, SpO_2_, respiratory rate, and stress metrics carry known measurement limitations documented in the device-validation literature. Nocturnal HRV from a comparable Garmin device showed only modest agreement with ECG reference in controlled validation (CCC = 0.87) [34], and Garmin’s SpO_2_ estimation has been shown to underestimate relative to reference pulse oximetry in a meaningful minority of readings (~16.7% below 95% when reference readings were ≥98%), while resting HR and short-window HRV under this same acquisition protocol (a 2-min Health Snapshot-style spot check) showed good agreement with reference instruments [35]. Comparable agreement for HR and HRV was also reported for a related Garmin device in a controlled polysomnography/ECG validation [36]. These findings motivate treating F-VRI’s inputs as noisy proxies for the underlying physiological states rather than as ground-truth measurements, consistent with the percentile-based fuzzification approach (Section 3.4), which is deliberately robust to this class of measurement noise rather than assuming precise, calibrated values.

The HRV metric reported throughout is RMSSD (root mean square of successive differences), the headline HRV value surfaced on Garmin’s standard Health Snapshot report; SDRR, a secondary detail metric in the same feature, was not used. Entropy weights (Section 3.4) were computed separately within each cohort using only that cohort’s own data, except for the cross-domain transfer analyses (Section 4.3), where weights fit on the source cohort were applied unmodified to the target cohort, as described in Section 3.6.

### 3.3. Design Rationale: A Wearable-Deployable, Label-Free Index

The F-VRI’s construct validity rests on a specific, well-documented branch of the vigilance-decrement literature: arousal- and drive-based accounts, which hold that sustained-attention decline under monotonous, low-event-rate conditions is accompanied by declining autonomic activation, distinct from, but complementary to, the resource-depletion account discussed in Section 2.1 [37]. Direct psychophysiological evidence supports this specifically for the underload regime: Pattyn et al. [38] recorded cardio-respiratory autonomic markers during a sustained-attention task designed explicitly to test the underload against the overload hypothesis of vigilance decrement, and found the physiological pattern—declining sympathetic activation—supported the underload account. This bidirectionality is an established organizing principle in the ATC vigilance literature specifically, not an assumption particular to this study: our own systematic review of vigilance modeling and prediction in ATC [39] identifies impairment as bidirectional, with underloaded controllers disengaging and overloaded controllers losing resolution, and notes that current monitoring approaches do not reliably distinguish between these states. That review also found that cardiac/HRV-based vigilance correlates are reported inconsistently in direction across the small existing ATC literature—one reason the present study’s explicit regime specification and empirical validation contribute new, targeted evidence to an open question rather than assuming a settled one. Both populations studied here (routine ATC shifts, simulator training sessions) are monotonous, low-event-rate monitoring contexts of the underload type; F-VRI’s cost/benefit construction (Section 3.4) is correspondingly oriented to detect rising activation as risk in general, while the underload regime studied here is expected, and empirically observed (Section 4.1), to show the index declining over a shift, consistent with autonomic withdrawal accompanying vigilance decrement under monotony. This direction is regime specific: under acute overload or stress, the same construct would be expected to rise, a boundary condition returned to in Section 5.4 and flagged for validation in higher-workload operational contexts in future work.

Two further terms used throughout this paper warrant the same precision. Strain, as used here, denotes the cumulative cost of sustained arousal regulation over a shift, rather than the instantaneous arousal level itself: two operators can show the same momentary arousal reading while differing in how much regulatory effort it took to sustain or suppress it, and F-VRI, built from single pre/post snapshots, does not distinguish these—it indexes momentary activation, and “strain” in its name signals the intended construct, not a separately measured quantity. Fatigue is distinct again: following the definition adopted in our own review of this literature [39], fatigue is a cumulative reduction in physiological or cognitive capacity, downstream of accumulated strain, that can manifest as vigilance decrement through either of two distinct pathways—underload-driven disengagement or overload-driven resource depletion—which is precisely why this paper treats fatigue as a possible cause of the observed pattern rather than as a construct F-VRI measures directly.

The target application is continuous, real-time vigilance/strain monitoring from the wrist-worn device while a controller is actively working, an IoT-style streaming deployment model consistent with the architectures reviewed in Section 2.6 [12,19]. This constrains model design in two respects not typical of laboratory fatigue or emotion-elicitation research. First, no behavioral ground truth is available at inference time: the PVT requires the operator to stop working and complete a multi-minute reaction-time battery, which is operationally infeasible during live traffic control, and no comparable continuous self-report of affective state is available either. PVT is therefore retained strictly as an offline validation benchmark that the deployable model must detect the underlying state change at least as reliably as, without requiring it as an input. Second, no continuous fatigue or affect labels exist for supervised model training; only two (or, for two trainees, four) labeled snapshots per individual are available, in a small overall sample. Supervised models trained under these conditions risk fitting domain-specific noise rather than a generalizable signal (evaluated directly in Section 4.3).

These constraints motivated an unsupervised, wearable-only fuzzy multi-criteria decision-making (MCDM) index, built without using pre/post labels, evaluated against both the PVT benchmark and a range of supervised machine learning baselines trained under a best-case protocol.

### 3.4. Fuzzy MCDM Vigilance/Strain Index

**Figure 2:**
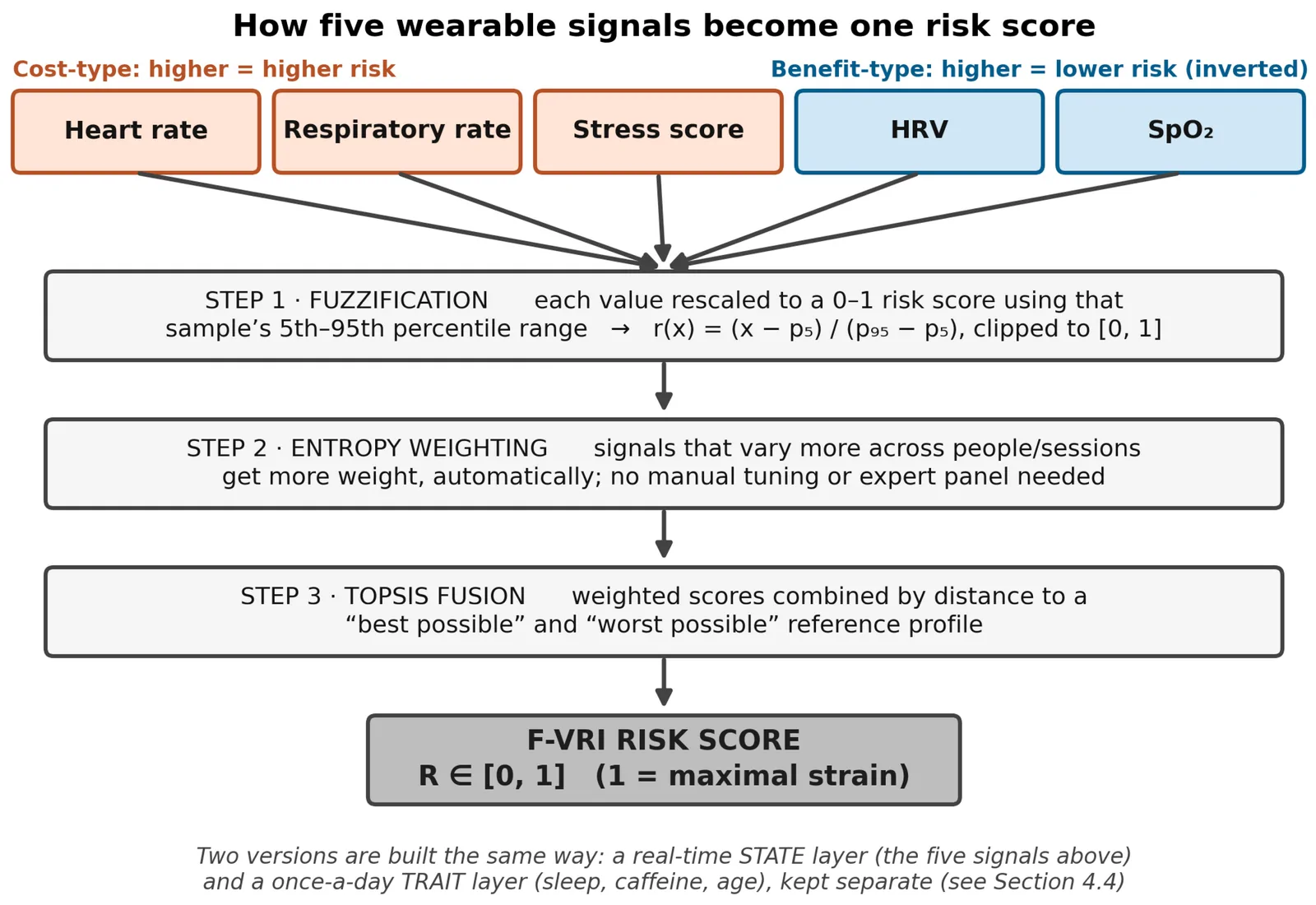
How the five wearable signals are combined into the F-VRI risk score. Orange boxes denote cost-type criteria (higher value = higher risk); blue boxes denote benefit-type criteria (higher value = lower risk), which are inverted before aggregation. Gray boxes show the processing steps and the resulting risk score.

The index is constructed in three steps (fuzzification, entropy weighting, and TOPSIS aggregation), summarized in Figure 2 and detailed below.

**Criteria and directionality.** The deployable (“state-layer”) index uses the five continuously wearable-derivable physiological criteria described in Section 3.2: heart rate, HRV, SpO_2_, respiratory rate, and stress. Each criterion was assigned an a priori risk direction: higher heart rate, higher respiratory rate, and higher stress were treated as higher-strain (“cost”-type criteria); higher HRV and higher SpO_2_ were treated as lower-strain (“benefit”-type criteria) [40] and inverted prior to aggregation. The respiratory-rate direction is a working assumption pending confirmation against ATC-specific physiology literature and is noted as a limitation (Section 6).

A separate trait-layer index was constructed from the three between-person covariates (sleep duration, caffeine dose, age) as a once-per-day baseline risk stratifier, kept structurally separate from the state layer for reasons developed in Section 4.4.

**Fuzzification.** Each criterion was converted to a triangular fuzzy risk membership in [0, 1], anchored at the sample 5th and 95th percentiles:

r(x) = clip((x − p_5_)/(p_95_ − p_5_), 0, 1) (1)

with r(x) inverted (1 − r(x)) for benefit-type criteria. Percentile-based anchoring was used in the absence of universally agreed clinical or operational thresholds for all criteria in this population; literature- or expert-derived anchors are a natural refinement for future work.

A sensitivity analysis across five anchor widths (min/max, 1st/99th, 5th/95th, 10th/90th, and interquartile 25th/75th) showed the simulator-cohort pre/post effect was stable and significant throughout (*p* = 0.002–0.015), whereas the field-cohort effect was more sensitive to anchor width (*p* = 0.019 at min/max, widening to *p* = 0.294 at the interquartile range), consistent with our characterization of the field-cohort result as pilot-level rather than confirmed (Section 4.1 and Section 6).

**Criteria weighting.** Objective Shannon entropy weighting [41] was used to derive criterion weights, avoiding the need for an expert-elicitation panel:

E_j_ = −k Σ_i_ p_ij_ ln(p_ij_),   k = 1/ln(n)   d_j_ = 1 − E_j_,   w_j_ = d_j_/Σd_j_ (2)

Criteria with greater discriminating variance across the sample receive proportionally higher weight. This choice follows evidence that entropy-derived weights improve the discriminative power of TOPSIS-based rankings relative to equal or purely subjective weighting schemes [42].

**Aggregation.** Weighted fuzzy memberships were aggregated via TOPSIS distance [24] to the ideal-best (zero risk) and ideal-worst (maximal risk) reference points:

R = d(best)/[d(best) + d(worst)] (3)

yielding a composite risk score R ∈ [0, 1], where R = 1 denotes maximal fused physiological strain. Note that this risk score is the complement of the conventional TOPSIS relative-closeness statistic (which measures closeness to the ideal-best, i.e., a safety rather than a risk score); this distinction was verified analytically and numerically during pipeline development to avoid an inverted risk direction.

### 3.5. Validation Design

Four complementary checks were used to separately probe group-level, individual-level, robustness, and generalization properties of the index:

1. **Group-level pre/post comparison**: Wilcoxon signed-rank test [43] on paired pre/post scores, with matched-pairs effect size dz [44] (mean difference divided by the standard deviation of the differences).
2. **Per-person directional concordance**: the proportion of individuals whose post-session score moved in the theoretically expected direction, included because group-level means can mask poor individual-level reliability, which is the operationally relevant unit for a deployed monitoring tool.
3. **Weight sensitivity analysis**: entropy weights were perturbed ±20% (uniform random, 500 iterations per dataset) and the resulting Spearman rank correlation with the base-weight ranking was recorded.
4. **Cross-context transfer**: weights (or, for ML baselines, trained model parameters) derived in one dataset were applied unmodified to the other, testing generalization from the controlled simulator setting to the field setting and vice versa, which approximates a realistic deployment pathway (calibrate under controlled conditions, deploy operationally).

Construct validity was additionally assessed, strictly offline, against PVT reaction time and, in the simulator cohort, the KSS; neither was used to build the index at any stage.

### 3.6. Machine Learning Baselines

Five supervised classifiers, logistic regression [45], an RBF-kernel support vector machine [46], random forest [47], gradient boosting [48], and k-nearest neighbors [49], were trained on the same five physiological criteria as the fuzzy index, to predict session position (pre = 0, post = 1), using:

**Leave-One-Subject-Out cross-validation (LOSO-CV)** within each dataset, so that no individual’s data appeared in both training and test folds, a deliberately generous, best-case protocol for the supervised models.

**Full-dataset cross-domain transfer**, each model was trained on the complete data from one setting and evaluated, without retraining, on the other setting, mirroring the realistic scenario of calibrating on simulator data before field deployment.

For the fuzzy index, entropy weights were fit on the source domain and applied unmodified to the target domain; in the originally reported transfer test, the 5th/95th percentile fuzzification anchors were recomputed on the target domain, since these were treated as a normalization step external to the weighting scheme rather than a fitted parameter. Because this still introduces target-distribution information into the test, we additionally report a stricter variant in Section 4.3 in which both the fuzzification anchors and the entropy weights are frozen from the source domain and applied entirely unmodified to the target domain.

All ML models were evaluated with the same paired Wilcoxon test and concordance metric as the fuzzy index, for direct comparability. Default scikit-learn [50] (version 1.5) hyperparameters were used throughout, with no manual tuning or deviation from library defaults for any of the five baseline classifiers.

The pre/post classification task is balanced by design (38 pre/38 post sessions in the field cohort; 15 pre/15 post in the simulator cohort), and each Leave-One-Subject-Out fold removes a matched pre/post pair together, preserving class balance within every training fold; no class-weighting or resampling was therefore applied.

### 3.7. Software

All analyses were performed in Python 3.11, using pandas 2.2 [51], NumPy 1.26 [52], SciPy 1.13, scikit-learn 1.5 [50], and openpyxl 3.1. Analysis code and de-identified data are available from the corresponding author upon reasonable request.

## 4. Results

### 4.1. Descriptive Pre/Post Change

In the field cohort, heart rate decreased significantly from pre- to post-shift (83.8 → 80.9 bpm, *p* = 0.008), as did subjective stress (45.0 → 39.5, *p* = 0.035), while HRV showed a non-significant upward trend (41.2 → 46.7 ms, *p* = 0.054). PVT reaction time slowed on average (378.8 → 401.3 ms) but this was not significant at the group level (*p* = 0.220). The simulator cohort (15 pairs) showed a parallel pattern—heart rate and stress declining, HRV trending upward—while PVT again showed a non-significant trend (531.3 → 500.7 ms, *p* = 0.379).

This pattern, physiological arousal indicators declining across a work period while the behavioral vigilance marker fails to reach group-level significance, is consistent with an underload/resource-theory account of vigilance decline under monotony rather than a stress-accumulation (overload) profile [1,2], and directly illustrates the practical motivation for this study: a single-trial PVT measurement is both too noisy and too operationally intrusive to serve as a reliable real-time indicator on its own.

### 4.2. Fuzzy Index Versus PVT

**Figure 3:**
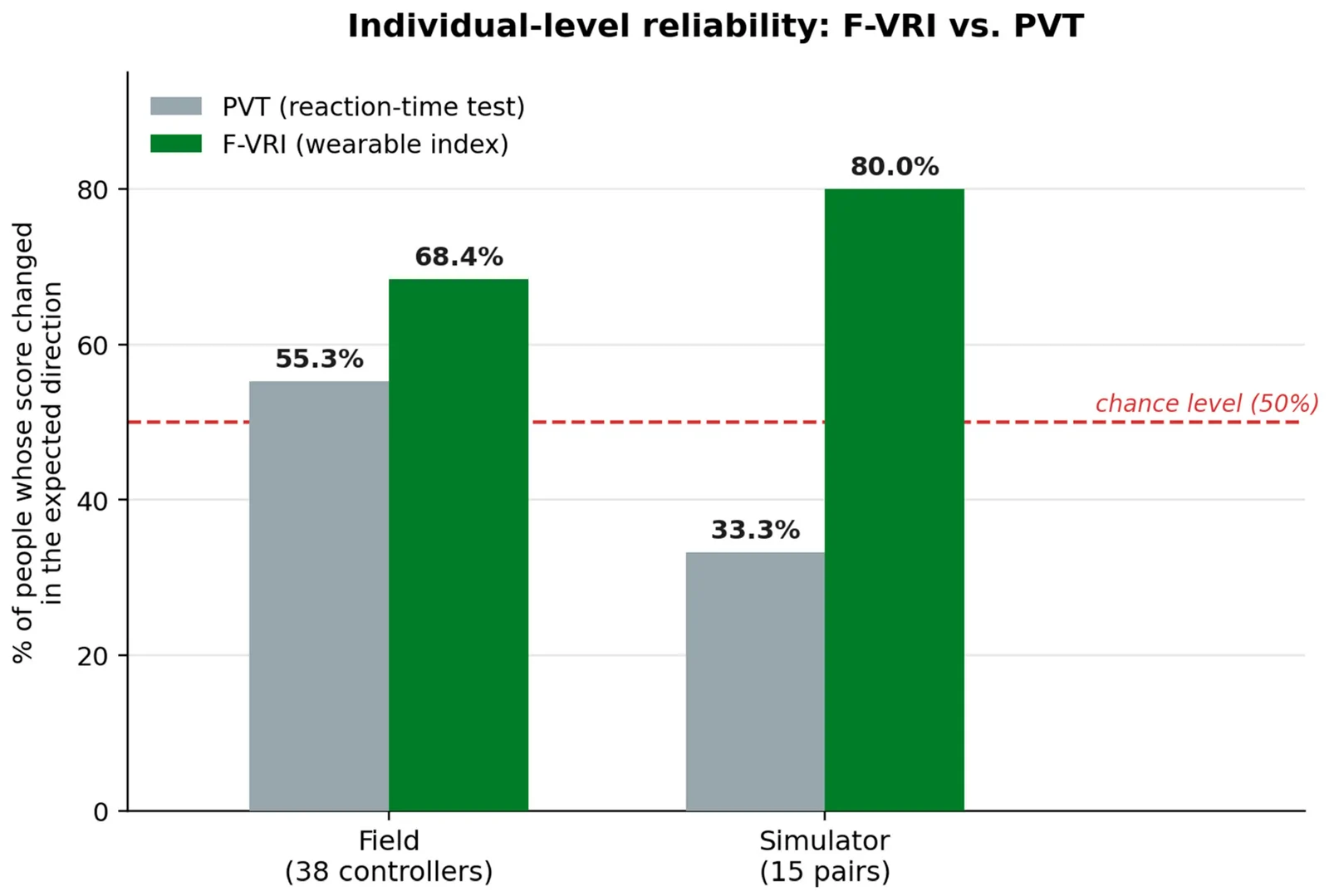
F-VRI showed higher directional concordance with the expected pre/post pattern than PVT in both cohorts, though an independent individual-level ground truth is absent; this should be read as a descriptive comparison rather than direct evidence of superior validity (Section 5.1).

Table 1 compares raw PVT and the fused fuzzy index at the group and individual levels, and Figure 3 shows the individual-level directional concordance for both cohorts.

The individual-level concordance results are the more operationally relevant finding: PVT performed at or below chance level for individual controllers/trainees in both settings: a single-trial reaction-time test is simply not a reliable indicator for any one person on any one occasion.

Offline construct-validity checks showed the fuzzy index correlated with PVT (r = 0.63, *p* < 0.001) and KSS (r = 0.54, *p* = 0.002) in the simulator cohort, where testing conditions were controlled, but not in the field cohort (r = −0.12, *p* = 0.28), plausibly reflecting the greater measurement noise of a single field PVT administration rather than a failure of the physiological index itself, though this remains an open question rather than a settled explanation.

Weight sensitivity analysis showed the index’s ranking of individuals was highly stable under ±20% weight perturbation (mean Spearman rank correlation = 0.987, field; 0.992, simulator), and cross-context transfer (weights fit on one setting, applied unmodified to the other) preserved a significant pre/post effect in both directions (field, *p* = 0.031; simulator, *p* = 0.022).

As a direct test of whether the index’s directional construction is doing necessary work, we compared it against a non-monotonic alternative in which each criterion’s fuzzy risk membership was defined symmetrically as distance from the sample median, rather than the directional cost/benefit mapping of Section 3.4. This symmetric formulation removed the pre/post effect entirely in both cohorts (field: *p* = 0.084 → 0.863; simulator: *p* = 0.004 → 0.389, dz reversing sign from −0.90 to +0.10), indicating that the detected shift-related change is not a generic magnitude-of-deviation artifact but specifically requires the directional assumption established in Section 3.3. This result demonstrates that, within the underload regime studied here, the assumed direction is doing genuine, falsifiable work rather than functioning as an arbitrary or interchangeable modeling choice.

### 4.3. Fuzzy Index Versus Machine Learning Baselines

**Figure 4:**
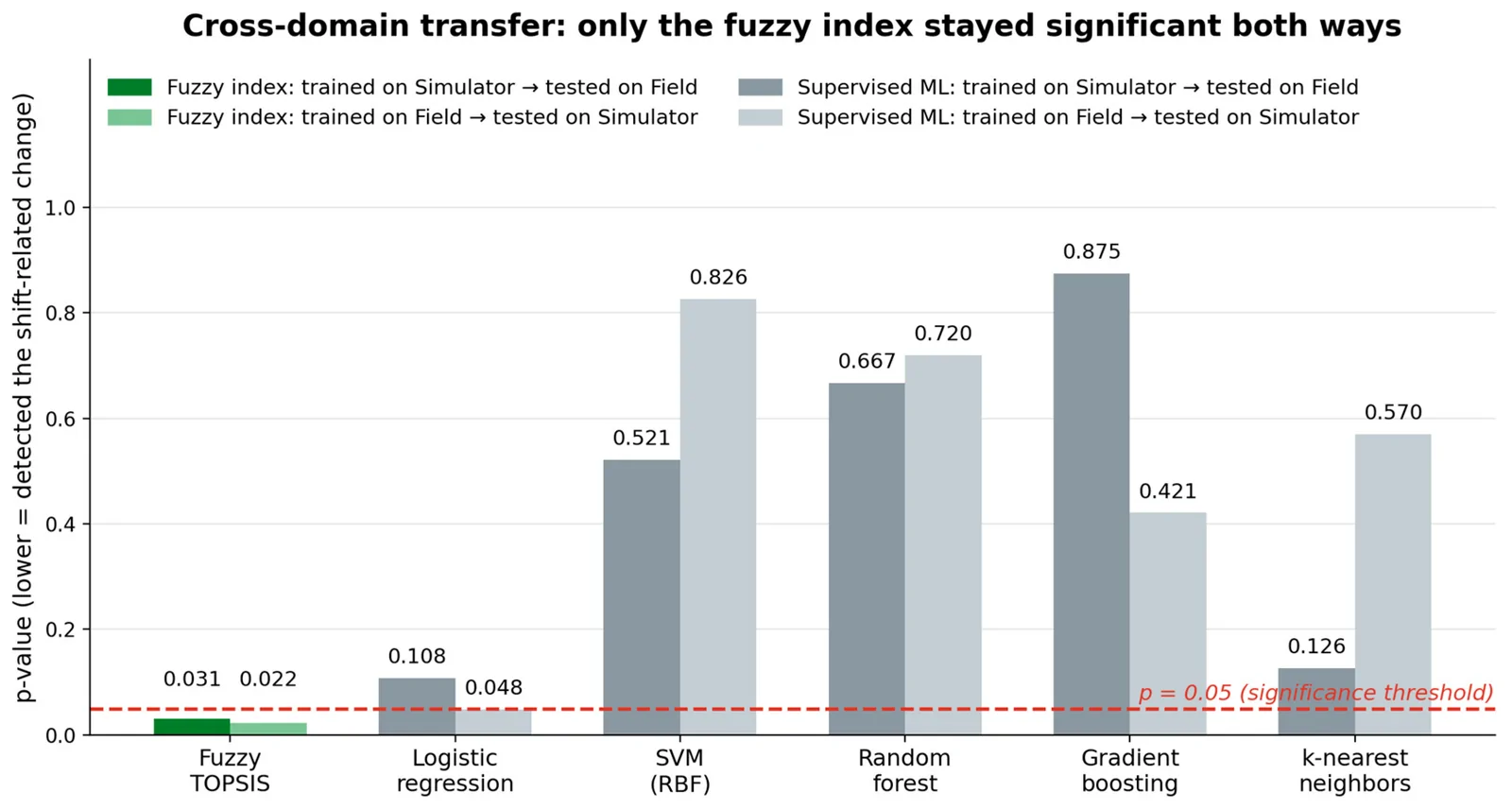
Cross-domain transfer: the fuzzy index was the only approach that stayed significant in both directions. Green bars show the fuzzy index and gray bars the supervised machine learning baselines; darker shades denote models trained on the simulator cohort and tested on the field cohort, and lighter shades the reverse direction. The dashed red line marks *p* = 0.05.

Table 2 reports within-domain detection for the fuzzy index and the five supervised baselines, while Table 3 and Figure 4 report the corresponding cross-domain transfer results.

Within a single domain, several supervised models occasionally matched or exceeded the fuzzy index (e.g., SVM on the field data, random forest on the simulator data), consistent with supervised methods’ ability to fit domain-specific structure when evaluated on the same domain they were trained on. However, the fuzzy index was the only model that remained significant, in a consistent direction, in both cross-domain transfer tests. Every supervised model failed to transfer in at least one direction, several collapsing toward a near-constant output under transfer (e.g., SVM: *p* = 0.826 on simulator-trained-on-field). A resampling stability check (50 iterations, 20% random subject dropout, simulator data) showed the fuzzy index’s effect size was consistently negative and moderate-to-large in magnitude (mean dz = −0.95, SD = 0.18); logistic regression showed comparable within-sample resampling variability (mean dz = +0.60, SD = 0.22) despite its poor cross-domain transfer performance, indicating that within-sample stability alone is an insufficient basis for judging real-world robustness and that cross-domain transfer testing is a necessary complement to it. This pattern is consistent with, and extends to a fully label-free comparator, the generalization gap increasingly reported when supervised wearable affect- and stress-recognition pipelines are tested outside their training distribution (Section 2.6) [3,6].

Because the originally reported transfer test recomputed the percentile fuzzification anchors on the target domain while freezing only the entropy weights (Section 3.6), we additionally ran a stricter transfer test in which both the fuzzification anchors and the entropy weights were frozen from the source domain and applied entirely unmodified to the target domain. The pre/post effect remained significant in both directions under this stricter protocol (field, trained on simulator: *p* = 0.045, dz = −0.36; simulator, trained on field: *p* = 0.010, dz = −0.74), closely comparable to the originally reported values (*p* = 0.031 and *p* = 0.022, respectively), indicating the cross-domain result is not an artifact of recomputing normalization anchors on the target domain.

### 4.4. A Weighting Pathology When Mixing Trait and State Criteria

**Figure 5:**
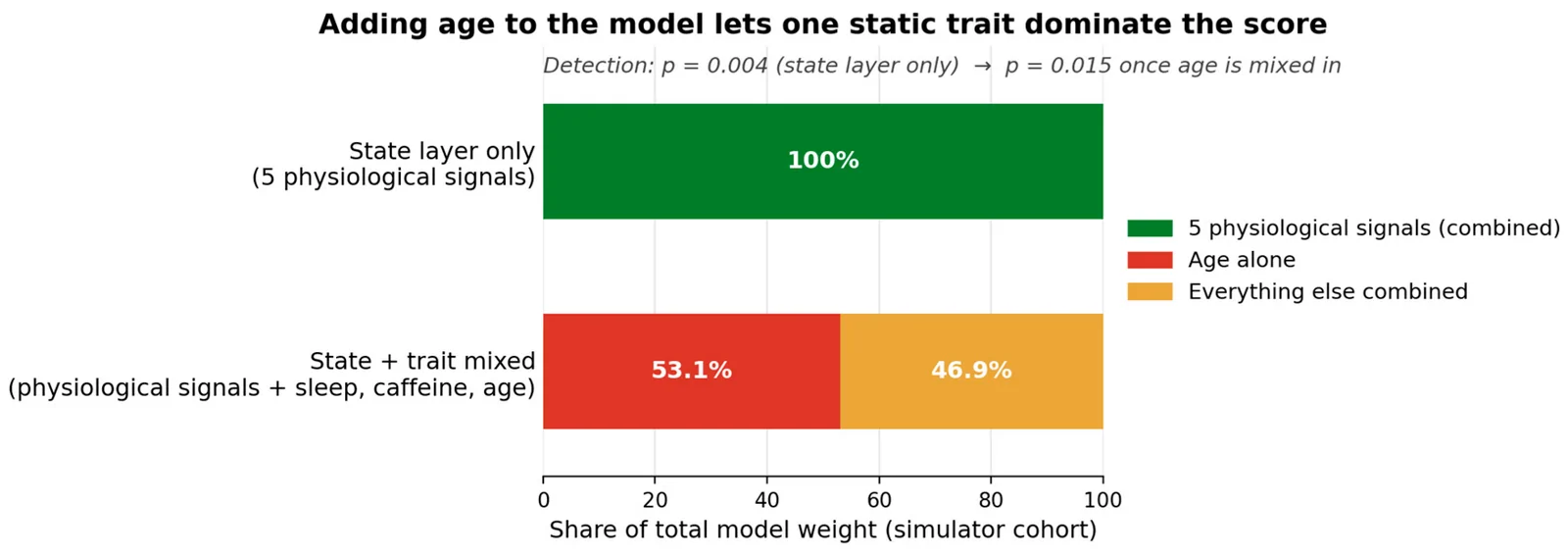
Mixing a static trait (age) into the model lets it dominate the weighting and weakens detection.

Figure 5 shows how the entropy weights are redistributed when static trait covariates are pooled with the five physiological criteria.

An exploratory extension incorporated the three between-person covariates (sleep duration, caffeine dose, age) into the same flat entropy-weighted fuzzy model as the five physiological criteria. This degraded detection performance relative to the state-only model (field: *p* = 0.084 → 0.111; simulator: *p* = 0.004 → 0.015, dz = −0.90 → −0.71). Inspection of the resulting entropy weights showed age alone received 53.1% of total model weight in the simulator sample and caffeine dose received 28.8% (field) to 14.9–19.2% (trait-only models) of weight across analyses. Because these covariates are, by construction, constant within each individual’s pre/post pair, they contribute between-person discriminability to the entropy calculation without contributing any within-person state-change signal; entropy weighting, being blind to this repeated-measures structure, systematically over-weights them at the expense of the dynamic physiological criteria that carry the actual pre/post signal.

This motivates a structural rather than a purely statistical fix: separating criteria into a state layer (dynamic, wearable-derived, appropriate for real-time detection) and a trait layer (static, appropriate for once-per-day baseline risk stratification, e.g., pre-shift fitness-for-duty flagging) rather than pooling both under a single weighting scheme. An exploratory test of whether trait-layer risk moderates the magnitude of in-shift state-layer change was not significant in either dataset (field: r = 0.12, *p* = 0.48; simulator: r = −0.38, *p* = 0.16, both likely underpowered at current sample sizes) and is reported as a null result rather than a supported finding.

This is not merely a description of what happened but a structural distinction with a direct empirical signature. Decomposing each criterion’s total variance into between-person and within-person components (a one-way random-effects decomposition) shows the five physiological criteria carry a real, nonzero within-person variance share (6.3–56.0% across criteria and cohorts), consistent with their capacity to register a pre/post state change, whereas the three static covariates (age, sleep duration, caffeine dose) show exactly 0.0% within-person variance in both cohorts, since each is recorded once and is by construction identical across a person’s pre/post pair. Entropy weighting is blind to this distinction—it rewards any criterion with high discriminating variance regardless of source—but the criteria themselves are not symmetric with respect to it: between-person variance in a state criterion can coincide with real within-person signal, while between-person variance in a trait criterion categorically cannot, since no within-person variance exists for entropy weighting to reward in the first place. This is why the same weighting logic is appropriate for the five physiological criteria but a documented pathology when static covariates are pooled into the same scheme.

A related independence concern applies within the state layer itself. The device-derived stress score is a proprietary algorithm output rather than a raw physiological measurement, and correlates near-perfectly with HR (Spearman r = 0.96 field, 0.97 simulator) and substantially with HRV (r = −0.67 field, −0.78 simulator), while SpO_2_ and respiratory rate are essentially uncorrelated with the other criteria and each other (|r| ≤ 0.20; full correlation matrices for both cohorts are given in Table 4). The five nominal criteria therefore behave as roughly three independent information dimensions rather than five: an {HR, stress} cluster, HRV, and SpO_2_/respiratory rate. This has two implications. First, for the auditability claim (Section 5.5): Stress is the one criterion whose internal computation is not inspectable, and given its near-redundancy with HR, its inclusion adds proprietary-algorithm opacity without adding independent information, weakening rather than strengthening the rule-legible framing. Second, for weighting: entropy weighting has no mechanism to detect or correct this redundancy, so the {HR, stress} cluster receives close to double the effective influence of a single independent physiological dimension. As a direct test, we rebuilt F-VRI with stress excluded (four criteria: HR, HRV, SpO_2_, respiratory rate). The simulator-cohort effect remained robust (*p* = 0.004 → 0.005, dz = −0.90 → −0.87), indicating the validated finding does not depend on this redundancy; the already pilot-level field-cohort effect weakened further (*p* = 0.084 → 0.180), indicating a non-trivial share of that marginal result was carried by the HR/stress duplication. We recommend either dropping stress as a criterion or replacing it with an independently-computed composite in future revisions of the index, and note this explicitly as a limitation of the present five-criterion construction.

## 5. Discussion

This section interprets the central findings reported in Section 4, relates them to the literature reviewed in Section 2, and considers their practical implications for ATC operator monitoring and for wearable-sensor psychophysiological state recognition more broadly.

### 5.1. Interpretation of the Central Finding

The central empirical finding of this study is that a label-free, wearable-only fuzzy index detects a shift-related physiological state change more reliably than the behavioral gold standard at the group level (Section 4.2), and shows higher directional concordance with the expected pattern at the individual level; the latter comparison, discussed below, should be read as descriptive rather than as independent proof of superior individual-level validity, since no independent ground truth for individual vigilance state is available in either cohort. This matters: PVT is validated precisely for its sensitivity to fatigue-related decline under controlled sleep-deprivation protocols [8], yet in the present operational and simulator settings, it performed at or below chance for individual controllers and trainees, while the fused physiological index showed acceptable-to-strong directional concordance in both cohorts. This divergence is plausibly explained by several non-exclusive factors rather than a single cause. First, PVT’s validated sensitivity was established under acute or chronic sleep-restriction protocols with large, controlled dose–response manipulations of fatigue, whereas the present shifts involve comparatively modest, naturally occurring physiological change that a single, once-per-session PVT administration may be poorly powered to detect at the individual level, compounded by the unknown occasion-to-occasion reliability of a single field PVT administration in this sample (Section 6). Second, a practice or familiarization effect is plausible, particularly among simulator trainees performing the PVT for one of their first times; a qualitative check on the two trainees contributing repeated blocks shows this pattern in one (PVT improving across blocks) but not the other, so we treat it as a plausible contributor rather than an established explanation. Third, regression to the mean may affect individuals whose pre-session PVT was unusually slow. Fourth, and most importantly, “below-chance” concordance presumes a single expected direction of change; without an independent, individual-level ground truth for vigilance, natural pre/post variation may in fact run in either direction for some individuals, so this pattern should not, on its own, be read as evidence that the fuzzy index is more valid than PVT (Section 5.2 returns to this point in the context of the machine learning comparison). The fuzzy index achieves this without ever using PVT or shift labels in its construction, which is an important distinction from the supervised machine learning comparison discussed next, and from the label-dependent supervised pipelines that dominate the wearable affective-computing literature reviewed in Section 2.6.

### 5.2. Interpretation of the Machine Learning Comparison

The machine learning comparison shows a specific, testable signature consistent with overfitting under small-N, label-scarce conditions: several supervised models were competitive with, or exceeded, the fuzzy index within the domain they were trained on, yet every supervised model failed to transfer, in at least one direction, when trained on one setting and tested unmodified on the other. Only the fuzzy index remained significant, in a consistent direction, under both cross-domain transfer tests. Within-domain competitiveness is the metric most commonly reported in the wearable fatigue- and affect-classification literature (e.g., [3,6,31]), and the present results suggest that model comparisons omitting an explicit domain-transfer test may overstate the operational readiness of supervised approaches for deployment in a new context. This caution is particularly relevant to small operational-safety datasets, where the number of labeled fatigue events is inherently limited and unlikely to grow substantially over time, and it echoes a generalization concern raised, but rarely tested directly, in recent reviews of wearable affective computing [6].

A related interpretive caveat deserves separate statement: predicting session position (pre vs. post) is not equivalent to predicting vigilance, fatigue, or strain. Any physiological variable that shifts systematically across a session for reasons unrelated to vigilance—adaptation to the sensor or task, circadian drift, postural change, hydration, ambient temperature, or sensor drift—could in principle support pre/post classification without tracking vigilance at all. The cross-domain transfer results (Section 4.3) therefore demonstrate that the fuzzy index’s pre/post-associated physiological pattern generalizes across domains more robustly than the supervised alternatives; they do not, on their own, demonstrate that this pattern specifically reflects vigilance rather than some other time-varying confound. This caveat applies with full force to the supervised machine learning baselines, which are trained on exactly this pre/post target and carry no independent link to vigilance beyond that label. It applies with less force, though not zero, to the fuzzy index specifically, because F-VRI carries convergent-validity evidence unavailable to the supervised baselines: its offline correlation with PVT (r = 0.63, *p* < 0.001) and KSS (r = 0.54, *p* = 0.002) in the simulator cohort (Section 4.2) ties it to two independently validated vigilance/sleepiness measures, providing a partial, though not complete, safeguard against the confound identified here. The cross-domain result is thus best read as evidence that the fuzzy index’s fused physiological signal generalizes better than the tested supervised alternatives, not as direct, standalone proof of superior vigilance-recognition transfer.

Relatedly, because the two cohorts differ in population, task, and setting simultaneously (Section 3.1), this cross-domain transfer result is best interpreted as a joint cross-population/cross-context stress test rather than as isolating simulator-to-field environmental generalization specifically.

### 5.3. Interpretation of the Trait/State Finding

The trait/state weighting pathology documented in Section 4.4, in which static between-person covariates absorbed a disproportionate share of entropy-derived model weight at the expense of the dynamic physiological signal, is, to the authors’ knowledge, a previously undocumented failure mode for objective (as opposed to expert-elicited) weighting schemes in fuzzy MCDM applications. It arises because entropy weighting rewards criteria with high discriminating variance across the sample, and static, between-person covariates such as age can carry substantial between-person variance while carrying no within-person state-change signal whatsoever; a purely statistical weighting rule, blind to this repeated-measures structure, cannot distinguish the two. The proposed two-layer state/trait architecture is offered as a general design pattern, not specific to vigilance or to ATC: any fuzzy MCDM application that naively pools dynamic, repeatedly measured criteria with static, between-person covariates under a single objective weighting scheme, including physiological arousal- or emotion-recognition systems that mix moment-to-moment autonomic signals with static demographic or trait covariates, is, in principle, susceptible to the same pathology, and should consider a comparable structural separation.

### 5.4. Relation to the Vigilance, Fuzzy MCDM, and Affective-Computing Literature

This work draws on three research literatures that have so far developed largely independently of one another. The underload/resource-theory account of vigilance decrement [1,2] explains why sustained ATC monitoring is expected to degrade measurably over time even without acute sleep deprivation, and is broadly consistent with the field-cohort pattern reported in Section 4.1, in which physiological arousal indicators declined significantly across a shift while the PVT failed to reach group-level significance, one profile consistent with gradual, underload-driven disengagement [1,2,39], but equally consistent with circadian decline across the shift, documented directly in a real ATC operational setting [10], habituation or adaptation to the measurement protocol, physiological recovery, or reduced anticipatory arousal as the shift progresses and concludes, a pattern documented in a comparable safety-critical shift-work population [53]. Without independent workload, traffic-complexity, or time-of-day data for the present field cohort (Section 6), this pattern cannot be mechanistically attributed to any one of these processes over the others, and the underload account should be read as one plausible hypothesis rather than a demonstrated mechanism. Separately, fuzzy MCDM methods have an established track record as a label-free, auditable framework for multi-criteria safety risk ranking in road traffic safety, from identifying the factors most strongly associated with accident rates [15] to prioritizing hazard locations for intervention using integrated fuzzy AHP-TOPSIS [16], and have recently been extended to physiological driver-stress recognition directly [17,18,19]. The present study extends this fuzzy MCDM paradigm to air traffic control, a structurally analogous safety-critical, human-in-the-loop traffic-management domain in which operator vigilance is itself a criterion that could plausibly be fused into a broader operational safety risk assessment alongside traffic-complexity, staffing, and environmental criteria in future work. Third, and most broadly, this work is a direct instance of the wearable-sensor affective-computing and emotion-recognition problem reviewed in Section 2.6: it fuses the same class of autonomic physiological signals used throughout that literature [3,4,5,6] into a continuous arousal/strain risk score, but does so with a label-free method rather than the supervised or deep-learning classifiers that currently dominate that field, and validates it, unusually for that literature, under an explicit cross-context transfer test. Situating operator vigilance within this joint fuzzy MCDM/affective-computing framing strengthens the case for treating human factors and psychophysiological indicators as a core, quantifiable input to safety-critical monitoring generally, and suggests that label-free, interpretable fusion methods merit broader consideration within sensor-based emotion- and stress-recognition research, not only within transportation-safety applications.

### 5.5. Practical and Operational Implications

Beyond the specific ATC application, the results point to a practical deployment pathway, consistent with the IoT-oriented real-time stress- and affect-monitoring architectures described in recent reviews [12,19]. This framing is explicitly prospective: the present validation is based entirely on discrete pre/post snapshots (Section 3.1), and none of the operational claims below have yet been empirically tested. A deployed system would in principle involve continuous state-layer scoring from a wrist-worn device during a shift, providing a real-time, interpretable risk score without requiring the controller to interrupt task performance; periodic (e.g., pre-shift) trait-layer scoring as a separate, once-per-day fitness-for-duty risk stratifier; and a rule-legible, auditable output (an explicit distance-based risk score with transparent, inspectable criterion weights) suitable for supervisor-facing decision support in a domain where opaque model outputs are difficult to justify operationally or to defend under safety audit. Beyond routine monitoring, this same auditable index could also serve a post-incident function: should a safety occurrence arise, the rule-legible risk score would allow investigators to re-anchor the controller’s behavior in objective, human-factors-based physiological evidence gathered at the time of the event, complementing rather than replacing conventional incident-investigation procedures with a transparent, data-driven account of operator state.

Realizing this pathway requires several validation steps not undertaken in this study: sliding-window/streaming validation on continuously sampled data rather than discrete snapshots; within-shift temporal sensitivity to acute, mid-shift events rather than only pre/post averages; test–retest reliability of the index itself across repeated administrations; false-alarm-rate analysis at operationally relevant thresholds; calibration stability over time and across devices; sensitivity and specificity against independent operational outcomes, which were not available in this study; and a dedicated evaluation of the ethical and human-factors consequences of continuous physiological monitoring of certified operators, including consent, data governance, and the risk of monitoring being used punitively rather than supportively. Until these steps are completed, the operational framing offered here should be read as a prospective design target motivated by the present results, not a validated deployment-ready capability.

## 6. Limitations

Sample sizes are small, particularly for the simulator cohort (13 trainees, 15 analyzable pairs, two of which are non-independent within-subject repeats); the field-cohort group-level effect for the deployable index did not reach conventional significance (*p* = 0.084) and should be treated as a promising but underpowered pilot result rather than a confirmed effect. Several criterion-direction assumptions (respiratory rate; caffeine dose treated as a proxy for anticipated/compensatory fatigue rather than a direct physiological harm signal; age treated as risk-increasing) are working hypotheses drawn from the general fatigue/shift-work literature rather than ATC-specific evidence, and should be revisited as this work is extended. Relatedly, because KSS was collected only in the simulator cohort for operational reasons (Section 3.2), the field-cohort construct-validity assessment rests on PVT alone, which limits direct cross-cohort comparison of construct validity and should be addressed in future field-compatible protocols (e.g., a single-item sleepiness rating brief enough for operational use). Field PVT was administered once per session and its individual-occasion reliability could not be independently assessed in this dataset, which limits interpretation of its weak correlation with the physiological index in that cohort specifically. Additionally, the field cohort’s physiological data were not accompanied by traffic complexity, sector workload, communication density, or time-of-day/circadian information, so the underload interpretation offered in Section 5.4 for the observed pre/post pattern cannot be mechanistically distinguished from circadian decline, habituation to the measurement protocol, or reduced anticipatory arousal as the shift concludes; collecting these contextual variables alongside physiological monitoring is a priority for future field validation. All validation reported here is based on two (or, for two trainees, four) static pre/post snapshots per individual; extending the index to fully continuous, sliding-window operation on streaming wearable data, rather than discrete pre/post comparison, is a necessary next step before any operational claims can be made, and would also bring the design closer to the continuous, real-time recognition setting targeted by the wearable affective-computing literature reviewed in Section 2.6. Relatedly, the F-VRI is deliberately scoped to a single arousal/strain dimension derived from autonomic physiological signals; it does not attempt to recognize discrete emotion categories or the valence dimension of affect, and readers seeking a general-purpose emotion-recognition system rather than a domain-specific arousal/strain risk index should note this scope limitation explicitly. Finally, the entropy-weighting pathology identified in Section 4.4, and the two-layer structural fix proposed in response to it, were developed post hoc on the same data used to demonstrate the problem; independent replication of both the pathology and the fix in a new sample, and in other physiological state-recognition applications beyond ATC, would strengthen this contribution considerably.

Reviewer feedback also correctly identifies that a single monotonic risk direction cannot, in principle, represent both underload and overload regimes within one deployed index, since the two are expected to move physiological activation in opposite directions (Section 3.3). Generalizing F-VRI beyond the underload regime validated here would require a state-dependent architecture—for example, a non-monotonic membership function centered on an individual- or population-specific optimal activation band, following the Maximal Adaptability Model of stress and sustained attention [54], in which risk increases with distance from the band in either direction, combined with an external workload or task-context signal to resolve which side of the band a given reading represents. Testing such an architecture requires data from an overload/high-workload regime (e.g., high-traffic-density shifts or induced time pressure), which neither present cohort provides; we flag this explicitly as a necessary extension before F-VRI could be deployed across the full underload-to-overload spectrum, rather than the underload-specific regime validated here.

## 7. Conclusions

This study developed and evaluated a wearable-deployable, label-free Fuzzy Vigilance/Strain Risk Index (F-VRI) for air traffic controllers across a live-operational field setting and a simulator-based training setting, a combination not previously reported in the fuzzy MCDM literature for ATC operator monitoring; confirmatory validation was achieved in the simulator cohort, while the field-cohort result remains preliminary, pilot-level evidence (Section 6). Relative to the PVT behavioral gold standard, the fused index detected the pre/post shift-related state change more reliably at the group level and showed higher directional concordance at the individual level, without ever using PVT or shift labels in its construction; the individual-level comparison should be read descriptively rather than as independent proof of superior validity, absent ground truth (Section 5.1). Relative to five supervised machine learning baselines evaluated under a deliberately generous within-domain protocol, the fuzzy index was the only approach that remained significant, in a consistent direction, under an explicit cross-domain transfer test, which points to the practical value of label-free, interpretable methods under the small-sample, label-scarce conditions typical of operational aviation safety research and, more broadly, of wearable-sensor affective- and psychophysiological-state recognition. The exploratory identification of a trait/state weighting pathology, and the two-layer structural architecture proposed to correct it, offer a transferable design lesson for fuzzy MCDM applications beyond this specific domain, including physiological emotion- and stress-recognition systems that combine dynamic autonomic signals with static demographic covariates. These results support fuzzy MCDM as a promising foundation for future continuous ATC vigilance monitoring, pending the streaming validation and operational-outcome evidence identified above as necessary next steps, and as a resilient, explainable, and implementable sensor-fusion architecture for safety-critical psychophysiological state recognition under uncertainty more generally. Several findings, in particular, the field-cohort group-level effect for the deployable index (*p* = 0.084) and the post hoc trait/state correction, remain pilot level and require replication in larger, independent cohorts. Concrete next steps include recruiting a larger field cohort powered to confirm the deployable index’s group-level effect; extending the index from discrete pre/post snapshots to genuinely continuous, sliding-window scoring on streaming wearable data within an IoT-style monitoring architecture; grounding the fuzzy membership functions in ATC-specific rather than general shift-work literature as more targeted evidence becomes available; independently replicating both the entropy-weighting pathology and the proposed trait/state architectural fix in a new sample; and evaluating whether the label-free fuzzy fusion approach generalizes to other wearable-sensor affective- and emotion-recognition applications where cross-context transfer, rather than within-domain accuracy, is the operationally relevant benchmark.

## Figures and Tables

**Table 1:** Group-level and individual-level comparison of raw PVT and the fused fuzzy index (F-VRI, state layer).

Measure	Field (n = 38)	Simulator (n = 15)
PVT—Wilcoxon *p*	0.220	0.379
F-VRI—Wilcoxon *p*	0.084	0.004 (dz = −0.90)
PVT—per-person concordance	55.3%	33.3% (below chance)
F-VRI—per-person concordance	68.4%	80.0%

**Table 2:** Within-domain detection (Leave-One-Subject-Out CV for ML models); same five physiological inputs for all models.

Model	Type	Field *p*	Field Concordance	Simulator *p*	Simulator Concordance
Fuzzy TOPSIS	Unsupervised, no labels	0.084	68.4%	0.004	80.0%
Logistic regression	Supervised	0.819	47.4% (chance)	0.030	80.0%
SVM (RBF)	Supervised	0.018	65.8%	0.397	46.7%
Random forest	Supervised	0.072	65.8%	0.022	80.0%
Gradient boosting	Supervised	0.455	55.3%	0.074	66.7%
k-nearest neighbors	Supervised	0.061	23.7% (below chance)	0.673	26.7%

**Table 3:** Cross-domain transfer (trained in full on one setting, tested unmodified on the other).

Model	Field (Trained on Simulator) *p*	Simulator (Trained on Field) *p*
Fuzzy TOPSIS	0.031	0.022
Logistic regression	0.108	0.048
SVM (RBF)	0.521	0.826
Random forest	0.667	0.720
Gradient boosting	0.875	0.421
k-nearest neighbors	0.126	0.570

**Table 4:** Spearman correlation matrix of the five F-VRI criteria, by cohort (Field: n = 76 sessions; simulator: n = 30 sessions).

Stress	Resp	SpO_2_	HRV	HR	Criterion	Cohort
0.96	−0.05	0.01	−0.65	1.00	HR	Field
−0.67	0.10	−0.09	1.00	−0.65	HRV	Field
0.04	0.17	1.00	−0.09	0.01	SpO_2_	Field
−0.03	1.00	0.17	0.10	−0.05	Resp	Field
1.00	−0.03	0.04	−0.67	0.96	Stress	Field
0.97	0.20	0.01	−0.79	1.00	HR	Simulator
−0.78	−0.05	−0.04	1.00	−0.79	HRV	Simulator
−0.03	−0.09	1.00	−0.04	0.01	SpO_2_	Simulator
0.14	1.00	−0.09	−0.05	0.20	Resp	Simulator
1.00	0.14	−0.03	−0.78	0.97	Stress	Simulator

## Data Availability

The data presented in this study are available on request from the corresponding author.
